# Supplementary material for: Targeting pathogenic CD8+ tissue-resident T cells with chimeric antigen receptor therapy in murine autoimmune cholangitis
Source: Nat Commun. 2024 Apr 5;15:2936. doi: 10.1038/s41467-024-46654-5 (PMC10997620; doi:10.1038/s41467-024-46654-5)
Supplement: Supplementary file 1 — Supplementary Information [file 41467_2024_46654_MOESM1_ESM.pdf]

Supplementary Information

Targeting pathogenic CD8$^+$ tissue-resident T cells with chimeric antigen receptor therapy in murine autoimmune cholangitis

Hao-Xian Zhu[1, 2, #], Shu-Han Yang[1, #], Cai-Yue Gao[3], Zhen-Hua Bian[1], Xiao-Min Chen[1, 2], Rong-Rong Huang[4], Qian-Li Meng[5], Xin Li[3], Haosheng Jin[6], Koichi Tsuneyama[7], Ying Han[8], Liang Li[3, *], Zhi-Bin Zhao[3, *], M. Eric Gershwin[9, *] and Zhe-Xiong Lian[2, *]

[#]These authors contributed equally: Hao-Xian Zhu and Shu-Han Yang.

*Corresponding authors: Zhe-Xiong Lian, e-mail: zxlian@gdph.org.cn; M. Eric Gershwin: email: megershwin@ucdavis.edu; Zhi-Bin Zhao, email: zzbin@mail.ustc.edu.cn; Liang Li: email: liliang@gdph.org.cn.

Supplementary materials:
Supplementary figures 1-6
Supplementary table 1-2

Supplementary figure 1.

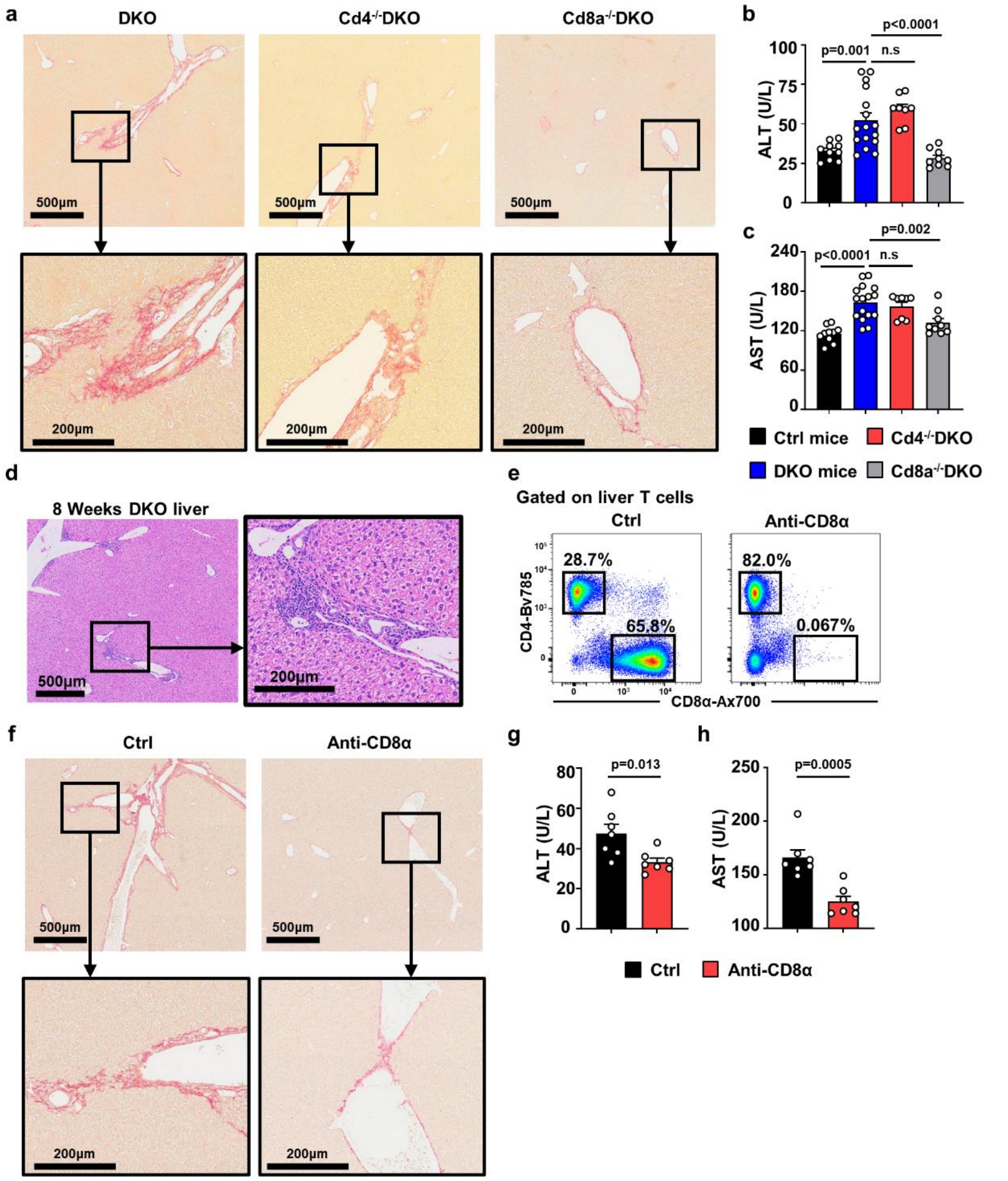

(a) Representative Sirius Red staining pictures of liver from DKO, Cd4$^{-/-}$DKO and Cd8$^{-/-}$DKO mice. Serum level of ALT (b) and AST (c) in Ctrl (n=10), DKO (n=16), Cd4$^{-/-}$DKO (n=8) and Cd8$^{-/-}$DKO (n=9) mice. (d) Representative H&E staining pictures of liver from DKO mice at the age of 8 weeks, magnification showing the portal area. (e) Representative FACS plot showing percentage of liver CD8^{+} T cells of DKO mice treated with the CD8α

depletion antibody or control IgG. (f) Representative Sirius Red staining pictures of liver from DKO mice treated with anti-CD8α depletion antibody and the control group. Serum level of ALT (g) and AST (h) in DKO treated with anti-CD8α depletion antibody and the control group. Data in (a, d, e, f) are representative results of at least 3 independent experiments. The P-values were determined by a one-way ANOVA with Tukey's multiple comparisons test (b. c), a two-tailed unpaired t-test (g, h). Data are shown as Means ± SEM.

Supplementary figure 2.

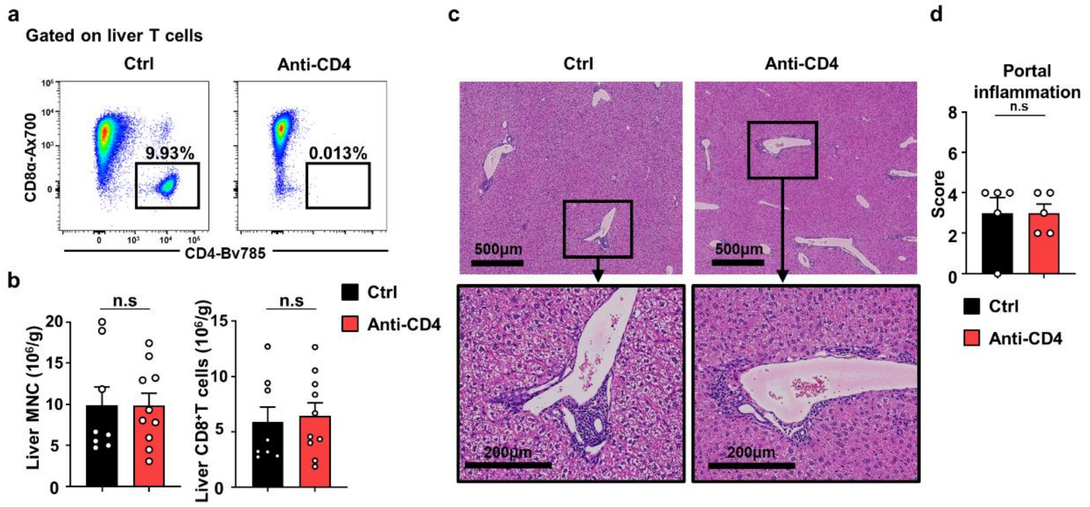

(a) Representative FACS plot showing the percentage of liver CD4⁺ T cells of DKO mice treated with the CD4 depletion antibody or control IgG. (b) Number of hepatic mononuclear cells and CD8⁺ T cells in DKO mice liver treated with the CD4 depletion antibody (n=10) or control IgG (n=8). (c) Representative H&E staining result of liver from DKO mice treated with the CD4 depletion antibody (n=10) or control IgG (n=8). (d) Pathological score of portal inflammation of DKO liver treated with anti-CD4 depletion antibody (n=5) and the control group (n=5). The P-values were determined by a two-tailed unpaired t-test (b). Data in (a, c) are representative results of at least 2 independent experiments. Data were shown as means ± SEM.

Supplementary figure 3.

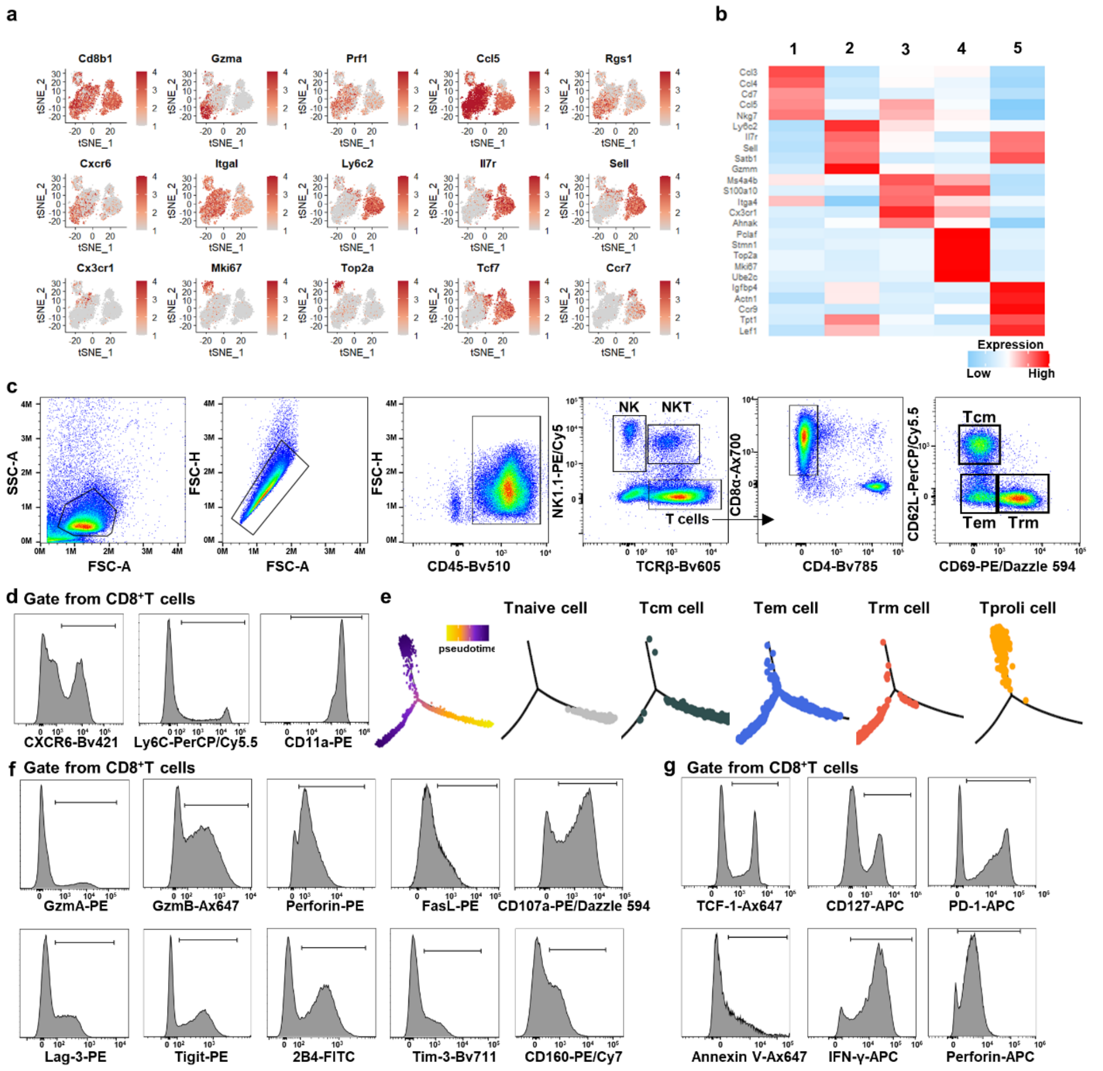

(a) Featureplot showing the expression of relevant genes in liver CD8⁺ T cells of DKO and WT mice. (b) Expression heatmap shows top 5 differentially expressed genes of each CD8⁺T cell cluster. (c) Flow cytometry gating strategy of DKO liver CD8⁺ T cells. (d) Representative flow cytometry gating strategy of CXCR6/ Ly6C in DKO liver CD8⁺ T cells. The histograms were gated from CD8⁺ T cells as descripted in (c). (e) Differentiation trajectory of CD8⁺ T cell subset by monocle analysis. (f) Representative flow cytometry gating strategy of GzmA/ GzmB/ Perforin/ FasL/ CD107a/ Lag3/ Tigit/ 2B4/ Tim-3/ CD160

in DKO liver CD8$^+$ T cells. The histograms were gated from CD8$^+$ T cells as descripted in (c). (g) Representative flow cytometry gating strategy of Tcf-1/ CD127/ Annexin V/ IFN-γ in DKO liver CD8$^+$ T cells. The histograms were gated from CD8$^+$ T cells as descripted in (c).

Supplementary figure 4.

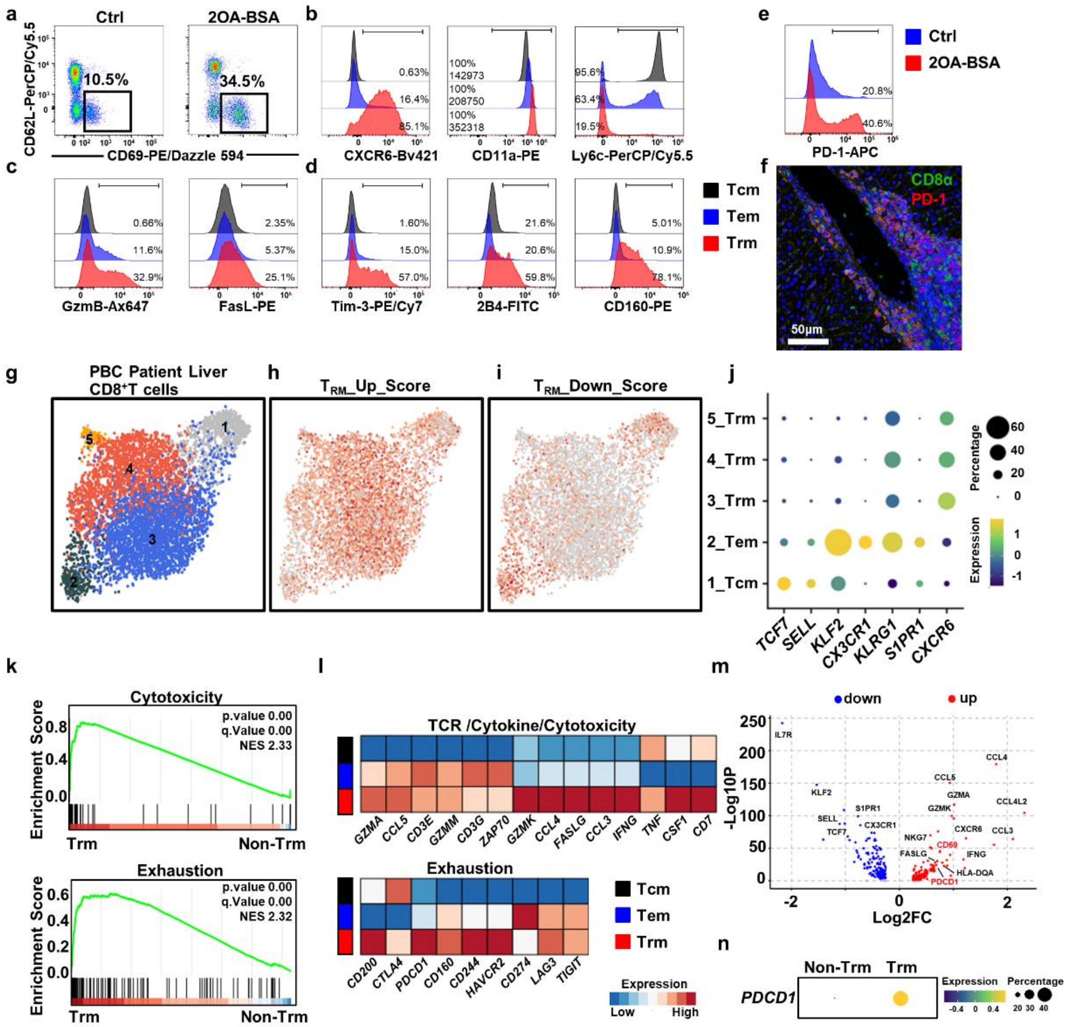

(a) Representative FACS plot showing the percentage of $CD69^+CD62L^-CD8^+$ T cell subset from liver of Ctrl mice or 2OA-BSA-induced PBC mice. (b) Representative FACS histogram of CXCR6, CD11a and Ly6C expression on $CD8^+$ Tcm, Tem and Trm cell subsets in the liver of 2OA-BSA-induced PBC mice. The gates indicating positive populations and their percentages of CXCR6, Ly6C, and gMFI of CD11a are shown in the figure. (c) Representative FACS histogram of Granzyme B and FasL expression on $CD8^+$ Tcm, Tem and Trm cell subsets in the liver of 2OA-BSA-induced PBC mice. The gates indicating

positive populations and their percentages of GzmB, and gMFI of FasL are shown in the figure. (d) Representative FACS histogram of selected immune-checkpoint molecule expression on $CD8^+$ Tcm, Tem and Trm cell subsets in the liver of 2OA-BSA-induced PBC mice. The gates indicating positive populations and their percentages of Tim3, 2B4, and gMFI of CD160 are shown in the figure. (e) Representative FACS histogram of PD-1 expression on $CD8^+$ Trm cell in the liver of Ctrl mice or 2OA-BSA-induced PBC mice. The gates indicating positive populations and their percentages are shown in the figure. (f) Representative multi-colored immunohistochemistry with DAPI (blue), CD8α (green) and PD-1 (red) of liver from 2OA-BSA-induced PBC mice. (g) UMAP plots of liver $CD8^+$ T cell clusters from PBC patients. (h, i) Feature plots showing the expression score of Trm up-or down-regulated genes by $CD8^+$ T cell clusters. (j) Dot plot showing the marker genes of $CD8^+$ T cell subsets. The color of dots represents the average expression of genes and size for the percent of cells expressing the genes. (k) The GSEA plots showing the enrichment score of cytotoxicity and exhaustion associated genes between $CD8^+$ Trm and non-Trm cells. (l) Heatmap showing the mRNA expression levels of TCR signaling, cytokine production, cytotoxicity and exhaustion associated genes by $CD8^+$ Tcm, Tem and Trm cells from liver of PBC patients. (m) Volcano plot showing the differentially expressed genes between $CD8^+$ Trm and non-Trm cells from liver of PBC patients. (n) Dot plot showing PD-1 expression by $CD8^+$ T cell subsets form liver of PBC patients. Experiments were repeated for 2 times. The P-values were determined by Wilcoxon test, Bonferroni p value correction (m).

Supplementary figure 5.

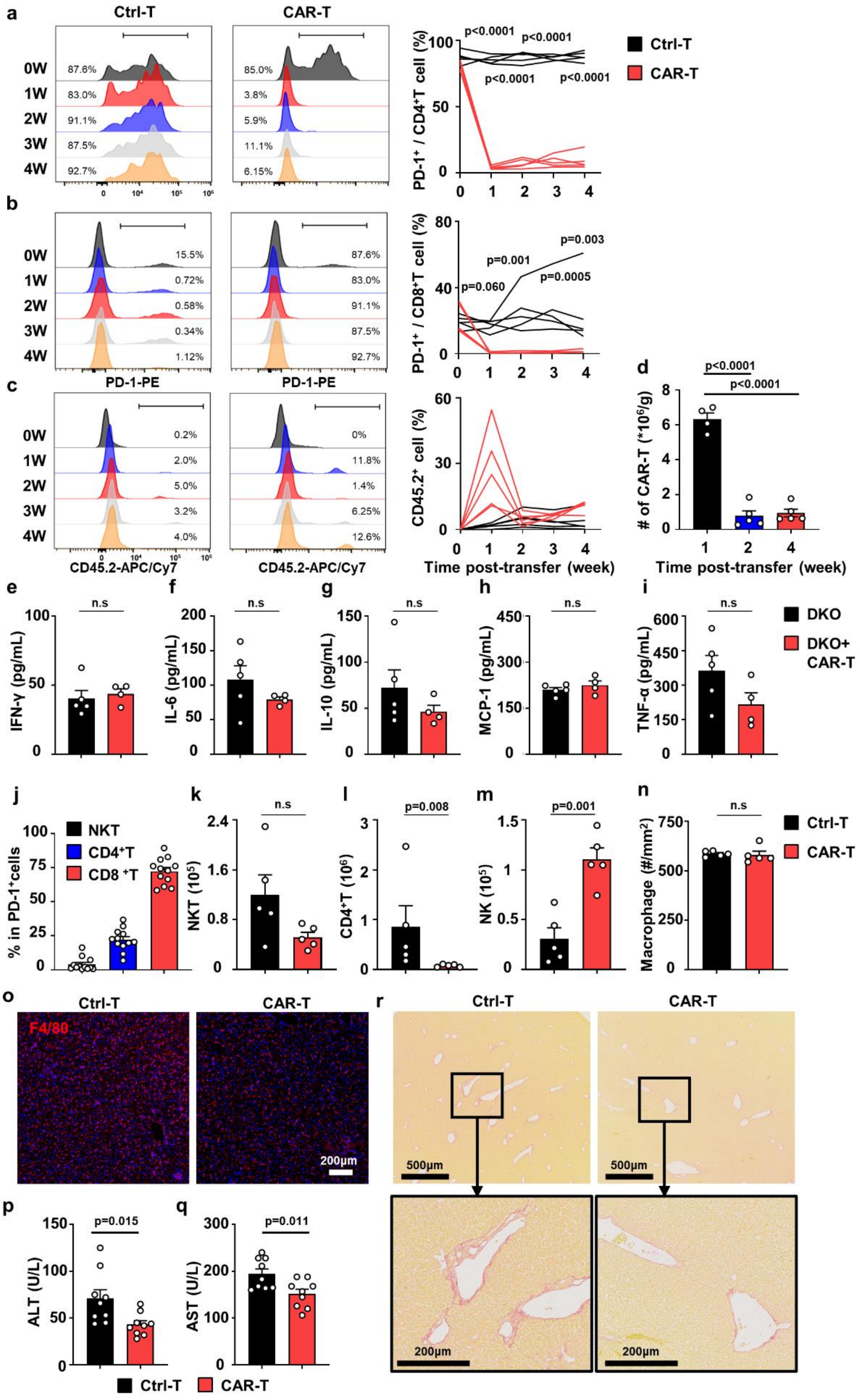

representative histogram and dynamics curves of percentage of PD-1^+CD4^+ T cells (a), PD-1^+CD8^+T cells (b) and CAR-T cells (c) in peripheral blood of DKO mice after PD-1 targeting CAR-T (n=5) or Ctrl-T infusion (n=5). The gates indicating positive populations and their percentages are shown in the figure. (d) The number of CAR-T cells in the liver of DKO mice at indicated time point after PD-1 targeting CAR-T infusion (n=5). (e-i) Serum levels of IFN-γ, IL-6, IL-10, MCP-1, TNF-α in DKO mice (n=5) and DKO mice infused CAR-T for 1week (n=4). (j) Percentages of NKT cells, CD4^+ T cells and CD8^+ T cells in PD-1^+ cells from DKO liver (n=12). Cell number of NKT cells (k), CD4^+ T cells (l) and NK cells (m) in DKO liver 4 weeks after PD-1 targeting CAR-T (n=5) or Ctrl-T infusion (n=5). Density of macrophage (n) and representative F4/80 immunohistochemistry staining (o) of DKO liver. Serum level of ALT (p) and AST (q) in DKO after PD-1 targeting CAR-T (n=5) or Ctrl-T infusion (n=5). (r) Representative Sirius Red staining pictures of liver from DKO liver 4 weeks after PD-1 targeting CAR-T (n=5) or Ctrl-T infusion (n=5). P values were assessed using 2-way ANOVA (a-c), 1-way ANOVA (d) and two-tailed unpaired t-test (e, f, g, h, i, k, l, m, n, p, q). Data in (a, b, c, o, r) are representative results of at least 2 independent experiments. Data were shown as means ± SEM.

Supplementary figure 6.

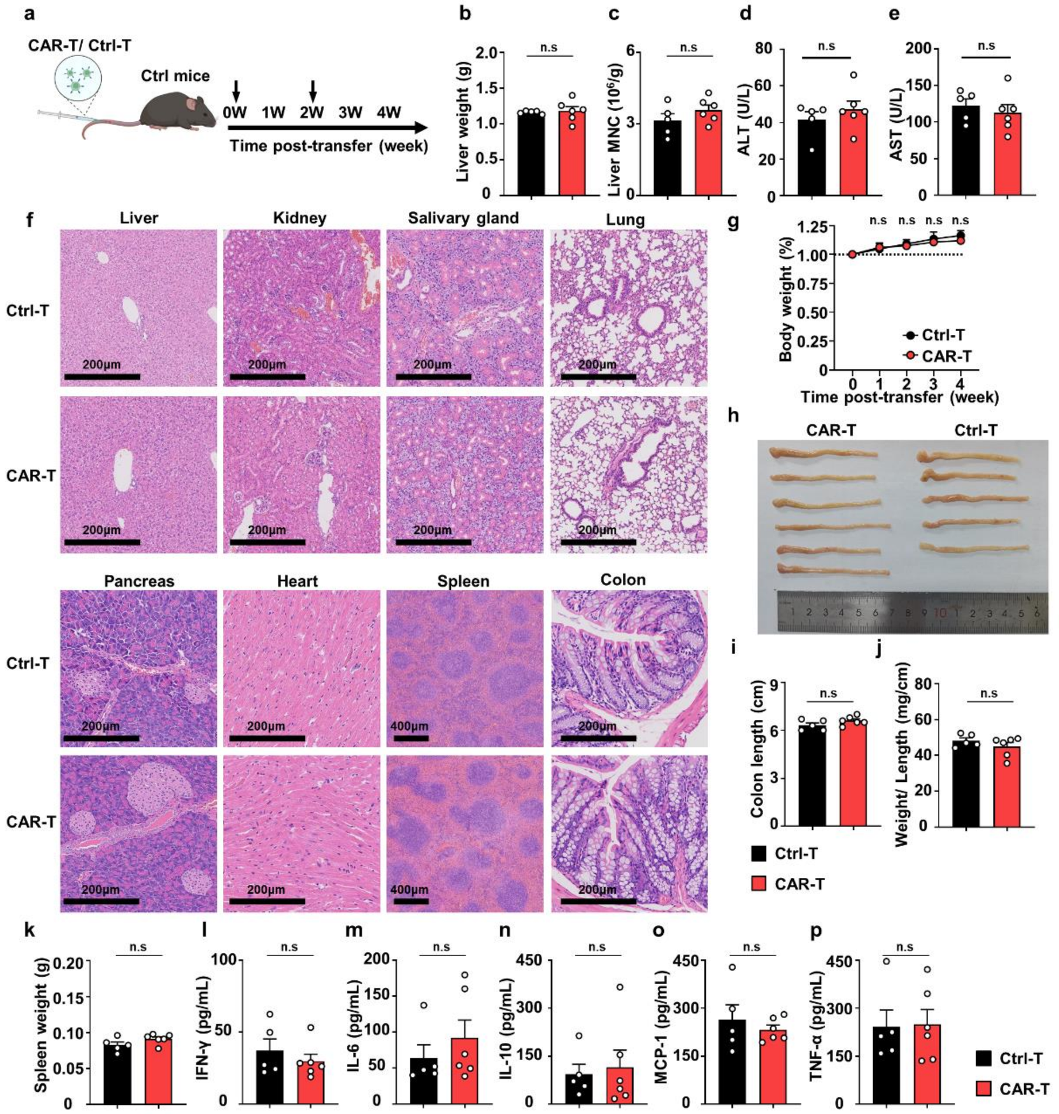

(a) The experimental procedure of anti-PD-1 CAR-T safety test. The diagram was created with BioRender.com. The liver weight (b) and number of liver MNCs (c) in control mice after PD-1 targeting CAR-T (n=6) or Ctrl-T infusion (n=5). Serum levels of ALT (d) and AST (e) in control mice after PD-1 targeting CAR-T (n=6) or Ctrl-T infusion (n=5). (f) Representative H&E staining pictures of liver/ kidney/ salivary gland/ lung/ pancreas/ heart/ spleen/ colon from control mice treated with PD-1 targeting CAR-T or Ctrl-T. (g) Dynamics curves of body weight of control mice after PD-1 targeting CAR-T (n=6) or Ctrl-T infusion (n=5). The

appearance of colons (h), colon length (i) and colon weight-length ratio (j) in control mice after PD-1 targeting CAR-T (n=6) or Ctrl-T infusion (n=5). (k) The weight of spleen in control mice after PD-1 targeting CAR-T (n=6) or Ctrl-T infusion (n=5). (k-p) Serum levels of IFN-γ, IL-6, IL-10, MCP-1, TNF-α in control mice after PD-1 targeting CAR-T (n=6) or Ctrl-T infusion (n=5). The P-values were determined by a two-tailed unpaired t-test (b-e, i-p) and 2-way ANOVA (g). Data in (f) are representative results of at least 2 independent experiments. Data are shown as Means ± SEM.

Supplementary table 1. Top50 differentially expressed genes in liver CD8^{+}T cells.

p_val	avg_log2FC	pct.1	pct.2	p_val_adj	cluster	gene
0	2.422235	0.847	0.331	0	0	Ccl4
0	2.360723	0.747	0.211	0	0	Ccl3
0	2.279547	0.94	0.62	0	0	Cd7
0	2.002115	0.848	0.304	0	0	Gzmb
0	1.614005	0.999	0.899	0	0	Ccl5
0	1.205784	0.996	0.93	0	0	Nkg7
2.99E-303	1.159238	0.982	0.884	5.07E-299	0	AW112010
2.16E-297	1.048492	0.98	0.937	3.67E-293	0	Cd3e
1.76E-282	1.204156	0.931	0.849	2.98E-278	0	Cd3g
4.53E-231	1.974831	0.743	0.417	7.69E-227	0	Rgs1
8.52E-227	0.907376	0.981	0.938	1.44E-222	0	Trac
3.46E-222	1.581291	0.728	0.352	5.87E-218	0	Tox
1.32E-213	1.335561	0.792	0.494	2.23E-209	0	Chsy1
2.67E-208	1.674134	0.547	0.162	4.53E-204	0	Lag3
2.94E-204	1.204356	0.783	0.345	4.99E-200	0	Gzmk
7.81E-199	1.463599	0.764	0.527	1.32E-194	0	Sh2d2a
5.25E-198	1.284331	0.86	0.66	8.91E-194	0	Id2
1.09E-194	0.572397	0.999	0.998	1.85E-190	0	H2.K1
2.45E-194	1.008172	0.922	0.839	4.15E-190	0	Cd8a
3.52E-186	1.749756	0.507	0.151	5.97E-182	0	Nr4a2
4.71E-181	1.121522	0.959	0.908	7.98E-177	0	Malat1
6.08E-	3.096414	0.524	0.177	1.03E-	0	Gzma

181				176		
1.03E-178	0.95254	0.919	0.84	1.75E-174	0	Fyn
4.57E-178	1.574111	0.387	0.072	7.75E-174	0	X2900026A02Rik
5.43E-174	1.308085	0.331	0.041	9.21E-170	0	Adgrg1
1.88E-171	0.876212	0.992	0.972	3.18E-167	0	Trbc2
2.06E-154	1.445467	0.476	0.165	3.49E-150	0	Cd160
3.98E-148	1.354069	0.41	0.109	6.74E-144	0	Pdcd1
4.52E-147	1.42378	0.546	0.245	7.67E-143	0	Cxcr6
1.95E-146	1.477921	0.602	0.305	3.30E-142	0	Serpina3g
3.93E-144	1.03604	0.775	0.535	6.66E-140	0	Gimap7
6.82E-141	0.412465	1	1	1.16E-136	0	B2m
1.34E-140	0.694273	0.967	0.944	2.27E-136	0	Ucp2
1.01E-136	0.713642	0.913	0.854	1.72E-132	0	Arl6ip1
3.68E-133	0.72541	0.941	0.923	6.24E-129	0	Mbnl1
4.21E-127	0.403195	0.999	0.999	7.14E-123	0	H2.D1
6.59E-122	1.285611	0.568	0.305	1.12E-117	0	Bhlhe40
5.64E-121	0.834755	0.865	0.772	9.57E-117	0	Itgal
7.12E-121	0.992	0.726	0.561	1.21E-116	0	Efhd2
3.64E-117	0.733325	0.911	0.884	6.17E-113	0	Stk17b
6.28E-112	0.680002	0.94	0.925	1.06E-107	0	Gimap3
1.39E-109	1.125634	0.622	0.403	2.35E-105	0	Prf1
1.67E-108	0.908939	0.772	0.643	2.84E-104	0	Runx3

7.37E-108	1.254619	0.435	0.184	1.25E-103	0	Ptger4
3.74E-107	0.725906	0.875	0.807	6.34E-103	0	Itgb2
4.92E-104	0.656244	0.905	0.857	8.33E-100	0	Gimap4
1.86E-101	1.15429	0.325	0.095	3.16E-97	0	Camk2n1
6.14E-101	1.127179	0.476	0.239	1.04E-96	0	Nedd9
1.02E-92	1.06862	0.595	0.419	1.74E-88	0	Lax1
1.88E-92	0.853645	0.731	0.626	3.19E-88	0	Akna
0	2.612027	0.984	0.219	0	1	Ly6c2
0	1.941224	0.971	0.258	0	1	Il7r
0	1.693008	0.974	0.299	0	1	Sell
0	1.518995	0.876	0.215	0	1	Satb1
0	1.507423	0.508	0.042	0	1	Gzmm
0	1.469662	1	0.877	0	1	Rps19
0	1.420802	0.997	0.799	0	1	Rpl36a
0	1.279591	1	0.972	0	1	Rps12
0	1.267849	0.718	0.129	0	1	Nsg2
0	1.244473	1	0.94	0	1	Rps20
0	1.182755	0.999	0.863	0	1	Npm1
0	1.153268	0.999	0.963	0	1	Rpl13
0	1.131411	0.999	0.934	0	1	Eef1b2
0	1.117766	0.994	0.792	0	1	Rpl35
0	1.116842	0.999	0.9	0	1	Rps18
0	1.114804	1	0.956	0	1	Rpl12
0	1.091452	1	0.996	0	1	Rpl23
0	1.089336	1	0.976	0	1	Rps28
0	1.088786	1	0.966	0	1	Rps5
0	1.061601	1	0.999	0	1	Rplp1
0	1.058578	1	0.992	0	1	Rpl39
0	1.052995	1	0.991	0	1	Rps16
0	1.04929	0.999	0.911	0	1	Rps7
0	1.027064	1	0.943	0	1	Rpsa
0	0.998913	1	0.996	0	1	Rps24
0	0.997729	1	0.983	0	1	Rps6
0	0.996304	0.999	0.943	0	1	Rplp0
0	0.989351	1	0.956	0	1	Rps26
0	0.983208	1	0.996	0	1	Rps29
0	0.971026	1	0.987	0	1	Rpl5
0	0.961695	0.999	0.968	0	1	Rpl23a

0	0.950431	1	0.934	0	1	Rpl32
0	0.941525	1	0.986	0	1	Rplp2
0	0.930823	1	0.989	0	1	Rps4x
0	0.928461	0.999	0.917	0	1	Rpl3
0	0.906984	1	0.967	0	1	Rps21
0	0.887744	1	0.985	0	1	Rps8
0	0.88015	1	0.954	0	1	Rpl18
0	0.876271	1	0.988	0	1	Rps9
0	0.87463	1	0.993	0	1	Rps3
0	0.868762	0.999	0.98	0	1	Rpl27a
0	0.848791	1	0.994	0	1	Rpl17
0	0.84501	1	0.967	0	1	Rps11
0	0.843913	1	0.967	0	1	Rpl6
0	0.839421	1	0.987	0	1	Rpl30
0	0.83612	1	0.984	0	1	Rpl18a
0	0.820905	1	0.962	0	1	Rpl4
0	0.805813	1	0.983	0	1	Rps27a
0	0.802317	1	0.984	0	1	Rpl9
0	0.780106	1	0.996	0	1	Rps23
0	0.777642	1	0.995	0	1	Rpl37a
0	0.706633	1	0.992	0	1	Rps14
0	0.687878	1	0.997	0	1	Rps13
0	0.592998	1	1	0	1	Eef1a1
2.52E-155	0.924694	0.992	0.938	4.27E-151	2	Ms4a4b
4.83E-146	1.063431	0.943	0.791	8.18E-142	2	S100a10
4.04E-144	1.172806	0.918	0.623	6.84E-140	2	Itga4
1.26E-121	1.364745	0.281	0.041	2.14E-117	2	Cx3cr1
1.98E-116	1.228141	0.869	0.652	3.36E-112	2	Ahnak
5.08E-107	1.104374	0.807	0.513	8.62E-103	2	Lgals1
3.92E-99	0.546506	1	1	6.64E-95	2	Actb
1.38E-92	0.794019	0.939	0.88	2.34E-88	2	Lsp1
4.86E-90	1.034996	0.752	0.518	8.24E-86	2	Spn
9.55E-90	1.011866	0.786	0.562	1.62E-85	2	Flna
7.04E-80	0.509756	0.992	0.983	1.19E-75	2	Laptm5
4.29E-76	1.256423	0.506	0.231	7.27E-72	2	S100a4
1.71E-66	0.722535	0.912	0.846	2.89E-62	2	Sp100
6.38E-63	0.733074	0.826	0.698	1.08E-58	2	Anxa6

7.60E-60	1.05418	0.373	0.151	1.29E-55	2	Rora
3.64E-59	0.54936	0.955	0.917	6.17E-55	2	Arhgdib
1.37E-57	0.693909	0.864	0.779	2.32E-53	2	Rap1b
3.94E-54	1.031363	0.785	0.66	6.68E-50	2	S100a6
1.05E-53	0.527427	0.986	0.979	1.78E-49	2	Actg1
2.26E-53	0.747754	0.741	0.586	3.84E-49	2	Prr13
9.37E-48	0.551776	0.912	0.875	1.59E-43	2	Selplg
5.15E-46	0.395026	0.992	0.98	8.73E-42	2	Rac2
6.39E-46	0.744077	0.655	0.471	1.08E-41	2	Emp3
4.61E-45	0.840103	0.646	0.491	7.81E-41	2	Ripor2
6.53E-45	0.700354	0.771	0.648	1.11E-40	2	S100a11
2.79E-42	0.431886	0.962	0.947	4.73E-38	2	Arpc2
3.04E-42	0.609661	0.881	0.742	5.15E-38	2	Atp1b3
4.61E-42	0.64069	0.789	0.668	7.81E-38	2	Crip1
9.62E-42	0.277386	1	0.999	1.63E-37	2	H2.D1
4.00E-39	0.357586	0.999	0.996	6.77E-35	2	Cfl1
8.93E-39	0.447739	0.971	0.927	1.51E-34	2	Ccl5
7.50E-37	0.566068	0.791	0.71	1.27E-32	2	Ms4a6b
8.31E-37	0.486358	0.929	0.887	1.41E-32	2	Thy1
1.13E-36	0.781948	0.518	0.348	1.92E-32	2	Cxcr3
4.31E-36	0.858566	0.309	0.151	7.30E-32	2	Slamf6
6.69E-36	0.672561	0.423	0.238	1.13E-31	2	Syt11
4.11E-35	0.719363	0.602	0.463	6.96E-31	2	S100a13
1.04E-34	0.352845	0.967	0.969	1.76E-30	2	Coro1a
5.56E-33	0.526554	0.763	0.683	9.43E-29	2	Tmem50a
2.19E-32	0.431925	0.87	0.799	3.71E-28	2	Lat
8.52E-32	0.793199	0.483	0.318	1.45E-27	2	Ifi27l2a
1.93E-31	0.396909	0.915	0.884	3.27E-27	2	Cdc42
6.47E-31	0.762064	0.261	0.118	1.10E-26	2	Lgals3
7.87E-31	0.640916	0.589	0.454	1.33E-26	2	Myo1f
3.86E-30	0.334792	0.989	0.986	6.55E-26	2	Cd52
4.61E-29	0.533119	0.752	0.669	7.82E-25	2	Sub1
7.16E-29	0.507218	0.672	0.463	1.21E-24	2	Gzmk
2.08E-27	0.648143	0.589	0.473	3.53E-23	2	Cd28
5.52E-27	0.406447	0.878	0.862	9.36E-23	2	Cnn2
6.27E-27	0.301442	0.974	0.977	1.06E-22	2	Pfn1
0	3.664756	0.896	0.021	0	3	Pclaf
0	3.612062	0.973	0.087	0	3	Stmn1
0	3.38575	0.852	0.093	0	3	Top2a
0	3.160931	0.919	0.07	0	3	Mki67
0	2.940685	0.682	0.023	0	3	Ube2c
0	2.765163	0.775	0.014	0	3	Birc5
0	2.423512	0.726	0.006	0	3	Ccna2

0	2.254079	0.699	0.021	0	3	Rrm2
0	2.119729	0.921	0.174	0	3	Lmnb1
0	2.064715	0.601	0.006	0	3	Nusap1
0	1.948555	0.653	0.012	0	3	Ccnb2
0	1.945805	0.782	0.111	0	3	Mcm5
0	1.914146	0.701	0.013	0	3	Cdk1
0	1.882751	0.663	0.021	0	3	Uhrf1
0	1.86997	0.744	0.025	0	3	Cks1b
0	1.860158	0.628	0.009	0	3	Tpx2
0	1.832346	0.721	0.065	0	3	Lig1
0	1.812365	0.763	0.082	0	3	Smc2
0	1.795031	0.653	0.016	0	3	Cdca8
0	1.760166	0.576	0.015	0	3	Cenpe
0	1.695745	0.474	0.007	0	3	Cenpf
0	1.693918	0.601	0.035	0	3	Prc1
0	1.652449	0.751	0.078	0	3	Rrm1
0	1.570519	0.659	0.009	0	3	Spc24
0	1.53708	0.599	0.011	0	3	Kif11
0	1.523661	0.701	0.077	0	3	Ncapd2
0	1.460548	0.495	0.003	0	3	Hmmr
0	1.394647	0.565	0.009	0	3	Kif15
0	1.385012	0.495	0.007	0	3	Plk1
0	1.366915	0.53	0.005	0	3	Ckap2l
0	1.357272	0.449	0.015	0	3	Cdc20
0	1.349823	0.586	0.017	0	3	Tacc3
0	1.332218	0.578	0.016	0	3	Ncapg
0	1.286092	0.565	0.026	0	3	Asf1b
0	1.266704	0.549	0.033	0	3	Fbxo5
0	1.25297	0.551	0.027	0	3	Tk1
0	1.22214	0.445	0.007	0	3	Prr11
0	1.199056	0.511	0.021	0	3	Cit
0	1.158325	0.505	0.016	0	3	Clspn
0	1.14475	0.399	0.006	0	3	Ccnb1
0	1.134215	0.578	0.038	0	3	Mad2l1
0	1.128638	0.468	0.004	0	3	Aurkb
0	1.112971	0.497	0.019	0	3	Diaph3
0	1.060549	0.491	0.007	0	3	Rad51
0	1.056138	0.439	0.006	0	3	Cdca3
0	1.030763	0.424	0.006	0	3	Knl1
0	1.023425	0.462	0.01	0	3	Ncaph
0	0.996091	0.362	0.001	0	3	Esco2
0	0.993202	0.435	0.006	0	3	Ncapg2
0	0.989735	0.395	0.004	0	3	Cep55

0	0.980549	0.428	0.003	0	3	Nuf2
0	0.962371	0.418	0.003	0	3	Bub1
0	0.947893	0.437	0.015	0	3	Dlgap5
0	0.910242	0.397	0.006	0	3	Depdc1a
0	0.902837	0.403	0.009	0	3	Spc25
0	0.876127	0.356	0.002	0	3	Nek2
0	0.854079	0.358	0.002	0	3	E2f8
0	0.847234	0.366	0.005	0	3	Sgo1
0	0.831044	0.368	0.001	0	3	Kif4
0	0.814658	0.328	0.001	0	3	Kif2c
0	0.798915	0.376	0.006	0	3	Cenph
0	0.74753	0.358	0.003	0	3	Cdca5
2.23E-164	1.982675	0.505	0.073	3.78E-160	4	Igfbp4
1.89E-131	1.904335	0.766	0.272	3.21E-127	4	Actn1
2.15E-120	1.189292	0.274	0.023	3.64E-116	4	Ccr9
6.36E-114	0.655828	1	0.999	1.08E-109	4	Tpt1
9.62E-113	1.719548	0.755	0.309	1.63E-108	4	Lef1
1.54E-100	0.677525	1	0.999	2.61E-96	4	mt.Cytb
3.39E-97	0.765708	1	0.999	5.76E-93	4	Rplp1
3.91E-97	1.317546	0.351	0.057	6.63E-93	4	Rgcc
4.95E-97	0.756377	1	0.988	8.39E-93	4	Rps8
5.61E-96	0.679544	1	0.996	9.51E-92	4	mt.Nd4
1.93E-95	0.756876	1	0.997	3.27E-91	4	Rps29
4.91E-94	0.727322	1	0.989	8.33E-90	4	Rps3a1
1.03E-93	0.732022	1	0.997	1.74E-89	4	Rps24
1.27E-91	1.313767	0.324	0.05	2.16E-87	4	Dapl1
1.76E-90	0.803229	1	0.981	2.99E-86	4	Rps28
8.20E-88	0.86992	1	0.954	1.39E-83	4	Rps20
5.44E-86	1.322962	0.452	0.113	9.23E-82	4	Als2cl
6.92E-85	0.800959	0.992	0.959	1.17E-80	4	Rpl35a
8.88E-84	0.716144	1	0.99	1.51E-79	4	Rpl5
5.64E-82	0.839373	0.995	0.928	9.57E-78	4	Rpl31
2.80E-80	0.666719	1	0.994	4.75E-76	4	mt.Nd2
5.51E-80	0.481038	1	1	9.35E-76	4	Eef1a1
1.27E-77	1.297664	0.777	0.369	2.16E-73	4	Satb1
4.31E-77	1.133584	0.918	0.453	7.31E-73	4	Sell
5.56E-76	0.745638	1	0.965	9.43E-72	4	Rps26

1.45E-75	1.424258	0.5	0.157	2.46E-71	4	Selenop
1.03E-72	0.644929	0.997	0.991	1.74E-68	4	Rps4x
4.80E-72	0.730974	1	0.952	8.14E-68	4	Rps25
1.57E-70	0.733699	1	0.948	2.65E-66	4	Eef1b2
2.09E-68	0.705175	1	0.966	3.55E-64	4	Rpl12
2.58E-68	0.603891	1	0.997	4.37E-64	4	Rpl23
2.30E-66	0.719859	0.995	0.93	3.90E-62	4	Rps7
4.43E-66	0.558651	1	0.994	7.50E-62	4	Rps3
3.04E-65	1.080767	0.867	0.424	5.15E-61	4	Il7r
3.09E-65	0.695533	1	0.936	5.24E-61	4	Tmsb10
3.24E-64	1.072797	0.846	0.457	5.49E-60	4	Tcf7
3.61E-64	0.592479	1	0.989	6.13E-60	4	Rplp2
1.03E-63	0.588175	0.997	0.987	1.74E-59	4	Rps27a
1.34E-62	0.882671	0.955	0.778	2.28E-58	4	Rps17
1.87E-60	0.702555	0.997	0.934	3.17E-56	4	Rpl8
2.66E-60	0.702688	0.997	0.957	4.51E-56	4	mt.Nd4l
3.98E-60	0.74545	0.995	0.904	6.75E-56	4	Rps19
2.73E-58	0.557088	1	0.988	4.62E-54	4	Rpl9
3.13E-58	1.025306	0.888	0.647	5.31E-54	4	Dgka
1.37E-57	0.444178	1	1	2.33E-53	4	mt.Atp6
1.82E-57	1.146645	0.41	0.129	3.08E-53	4	Fam241a
1.49E-56	1.257881	0.479	0.174	2.52E-52	4	Rflnb
2.07E-56	0.599949	1	0.97	3.51E-52	4	Rpl4
6.13E-56	1.214667	0.484	0.184	1.04E-51	4	Ccr7
6.17E-56	0.532036	1	0.991	1.05E-51	4	Rps9

Supplementary table 2. Primer sequences for qRT-PCR

Acta2-F	AAGAGCATCCGACACTGCTGAC
Acta2-R	AGCACAGCCTGAATAGCCACATAC
Col1a1-F	CAGGGTATTGCTGGACAACGTG
Col1a1-R	GGACCTTGTTTGCCAGGTTCA
Tgfb1-F	GTGTGGAGCAACATGTGGAACTCTA
Tgfb1-R	CGCTGAATCGAAAGCCCTGTA
Timp1-F	TGAGCCCTGCTCAGCAAAGA
Timp1-R	GAGGACCTGATCCGTCCACAA
Ifng-F	TAGCCAAGACTGTGATTGCGG
Ifng-R	AGACATCTCCTCCCATCAGCAG
Ccl2-F	TAAAAACCTGGATCGGAACCAAA
Ccl2-R	GCATTAGCTTCAGATTTACGGGT
Tnfa-F	AAGCCTGTAGCCCACGTCGTA
Tnfa-R	AGGTACAACCCATCGGCTGG
S1pr1-F	GTGTAGACCCAGAGTCCTGCG
S1pr1-R	AGCTTTTCCTTGGCTGGAGAG
Hobit-F	CTCAGCCACTTGCAGACTCA
Hobit-R	CTGTCGGTGGAGGCTTTGTA
Hprt-F	AGGTTGCAAGCTTGCTGGT
Hprt -R	TGAAGTACTCATTATAGTCAAGGGCA
