# Supplementary material for: Targeting pathogenic CD8+ tissue-resident T cells with chimeric antigen receptor therapy in murine autoimmune cholangitis
Source: Nat Commun. 2024 Apr 5;15:2936. doi: 10.1038/s41467-024-46654-5 (PMC10997620; doi:10.1038/s41467-024-46654-5)
Supplement: Supplementary file 3 — Reporting Summary [file 41467_2024_46654_MOESM3_ESM.pdf]

nature portfolio

Corresponding author(s): M. Eric Gershwin

Last updated by author(s): Feb 13, 2024

Reporting Summary

Nature Portfolio wishes to improve the reproducibility of the work that we publish. This form provides structure for consistency and transparency in reporting. For further information on Nature Portfolio policies, see our Editorial Policies and the Editorial Policy Checklist.

Statistics

For all statistical analyses, confirm that the following items are present in the figure legend, table legend, main text, or Methods section.

n/a	Confirmed	
☐	☒	The exact sample size (*n*) for each experimental group/condition, given as a discrete number and unit of measurement
☐	☒	A statement on whether measurements were taken from distinct samples or whether the same sample was measured repeatedly
☐	☒	The statistical test(s) used AND whether they are one- or two-sided<br>*Only common tests should be described solely by name; describe more complex techniques in the Methods section.*
☒	☐	A description of all covariates tested
☐	☒	A description of any assumptions or corrections, such as tests of normality and adjustment for multiple comparisons
☐	☒	A full description of the statistical parameters including central tendency (e.g. means) or other basic estimates (e.g. regression coefficient) AND variation (e.g. standard deviation) or associated estimates of uncertainty (e.g. confidence intervals)
☐	☒	For null hypothesis testing, the test statistic (e.g. *F*, *t*, *r*) with confidence intervals, effect sizes, degrees of freedom and *P* value noted<br>*Give P values as exact values whenever suitable.*
☒	☐	For Bayesian analysis, information on the choice of priors and Markov chain Monte Carlo settings
☒	☐	For hierarchical and complex designs, identification of the appropriate level for tests and full reporting of outcomes
☒	☐	Estimates of effect sizes (e.g. Cohen's *d*, Pearson's *r*), indicating how they were calculated

Our web collection on statistics for biologists contains articles on many of the points above.

Software and code

Policy information about availability of computer code

Data collection	Cellular phenotype data was collected on a BD LSRFortessa cytometer running FACSDiva version 8.0.<br>RNA-seq data was collected on illumina HIseq6000 platform (Novogene, China)<br>scRNA-seq data was collected on BD Rhapsody<br>IHC Slides were imaged and whole slides scanned using a Vectra Polaris Automated Quantitative Pathology Imaging System (Akoya Biosciences)
Data analysis	Statistical analysis was performed using PRISM 8 (GraphPad Software LLC). Flow cytomeyery data was analyzed using FlowJO software v10 (FlowJo LLC). Bulk RNA-seq data was analyzed using DEseq2 (https://bioconductor.org/packages/release/bioc/html/DESeq2.html) and clusterProfiler.Single cell RNA-seq data analysis was performed using Seurat package (version 4.0.1). IHC quantify was performed using HALO software (Indica Labs).

For manuscripts utilizing custom algorithms or software that are central to the research but not yet described in published literature, software must be made available to editors and reviewers. We strongly encourage code deposition in a community repository (e.g. GitHub). See the Nature Portfolio guidelines for submitting code & software for further information.

Data

Policy information about availability of data

All manuscripts must include a data availability statement. This statement should provide the following information, where applicable:

- Accession codes, unique identifiers, or web links for publicly available datasets
- A description of any restrictions on data availability
- For clinical datasets or third party data, please ensure that the statement adheres to our policy

The RNA sequencing data in this study were deposited in the Gene Expression Omnibus database under accession number GSE241014 [https://www.ncbi.nlm.nih.gov/geo/query/acc.cgi?acc=GSE241014].
Singe-cell RNA sequencing data of DKO mice is available in the NCBI Gene Expression Omnibus under accession number GSE186333 [https://www.ncbi.nlm.nih.gov/geo/query/acc.cgi?acc=GSE186333].
Singe-cell RNA sequencing data of PBC patients is available in Genome Sequence Archive under accession number HRA002347 [https://ngdc.cncb.ac.cn/gsa-human/browse/HRA002347].
The code is availablecan be found at from the https://github.com/zhuhx97/DKO.git.
Source data are provided with this paper.

Research involving human participants, their data, or biological material

Policy information about studies with human participants or human data. See also policy information about sex, gender (identity/presentation), and sexual orientation and race, ethnicity and racism.

Reporting on sex and gender	N/A
Reporting on race, ethnicity, or other socially relevant groupings	N/A
Population characteristics	N/A
Recruitment	N/A
Ethics oversight	N/A

Note that full information on the approval of the study protocol must also be provided in the manuscript.

Field-specific reporting

Please select the one below that is the best fit for your research. If you are not sure, read the appropriate sections before making your selection.

☒ Life sciences ☐ Behavioural & social sciences ☐ Ecological, evolutionary & environmental sciences

For a reference copy of the document with all sections, see nature.com/documents/nr-reporting-summary-flat.pdf

Life sciences study design

All studies must disclose on these points even when the disclosure is negative.

Sample size	No statistical methods were used to determine sample size. Numbers of mice per group within each independent experiment were limited to numbers typically used in the field. For in vivo experiments of DKO mcie, we selected the number of mice according to our previous experience (PMID: 35300072, 30035379, 29307587, 29868034, 24651036).Likewise, in vitro experiments employed group sizes based on prior knowledge of variation,with three biological replicates utilized (PMID: 34716308, 30874628). The number of independent experiments and biological replicates was indicated in each figure legend.
Data exclusions	No data were excluded from data set.
Replication	Experiments were performed multiple times independently, as described in the figure legends.
Randomization	animals were aged-matched in experiments and randomly divided into different groups.
Blinding	Blinding is not relevant to our study as the investigators need to be aware of the genotypes of the cell lines and mouse strains.

Reporting for specific materials, systems and methods

We require information from authors about some types of materials, experimental systems and methods used in many studies. Here, indicate whether each material, system or method listed is relevant to your study. If you are not sure if a list item applies to your research, read the appropriate section before selecting a response.

Materials & experimental systems

n/a	Involved in the study
☐	☒ Antibodies
☐	☒ Eukaryotic cell lines
☒	☐ Palaeontology and archaeology
☐	☒ Animals and other organisms
☒	☐ Clinical data
☒	☐ Dual use research of concern
☒	☐ Plants

Methods

n/a	Involved in the study
☒	☐ ChIP-seq
☐	☒ Flow cytometry
☒	☐ MRI-based neuroimaging

Antibodies

Antibodies used	All antibodies used are commercially available and extensively used. Perforin-APC(Clone S16009A ,Cat#154303 ,BioLegend); PD-1-APC(Clone 29F.1A12 ,Cat#135209 ,BioLegend); Ly-6C-APC(Clone HK1.4 ,Cat#128015 ,BioLegend); IFN-γ-APC(Clone XMG1.2 ,Cat#505809 ,BioLegend); CD127 (IL-7Ra?-APC(Clone A7R34 ,Cat#135011 ,BioLegend); Ly-6C-APC/Cy7(Clone HK1.4 ,Cat#128025 ,BioLegend); CD45.2-APC/Cy7(Clone 104 ,Cat#109823 ,BioLegend); TCF1-Ax647(Clone C63D9 ,Cat#6709S ,CST); GZMB-Ax647(Clone QA16A02 ,Cat#372219 ,BioLegend); Annexin V-Ax647(Clone ,Cat#640911 ,BioLegend); CD8a-Ax700(Clone 53-6.7 ,Cat#100729 ,BioLegend); CD69-BV421(Clone H1.2F3 ,Cat#104527 ,BioLegend); CD45.2-BV510(Clone 104 ,Cat#109837 ,BioLegend); CD45.1-BV510(Clone A20 ,Cat#110741 ,BioLegend); TCRb-BV605(Clone H57-597 ,Cat#109241 ,BioLegend); CD44-BV711(Clone IM7 ,Cat#103057 ,BioLegend); CD8a-BV785(Clone 53-6.7 ,Cat#100749 ,BioLegend); CD4-BV785(Clone RM4-5 ,Cat#100551 ,BioLegend); CD44-FITC(Clone IM7 ,Cat#103021 ,BioLegend); CD244.2-FITC(Clone m2B4 (B6)458.1 ,Cat#133503 ,BioLegend); PD-1-PE(Clone 29F.1A12 ,Cat#135205 ,BioLegend); CD200R (OX2R)-PE(Clone OX-110 ,Cat#123907 ,BioLegend); CD178 (FasL)-PE(Clone MFL3 ,Cat#106605 ,BioLegend); CD11a-PE(Clone M17/4 ,Cat#101107 ,BioLegend); GZMA-PE(Clone 3G8.5 ,Cat#149704 ,BioLegend); Perforin-PE(Clone S16009A ,Cat#154305 ,BioLegend); Lag-3 (CD223)-PE(Clone C9B7W ,Cat#12-2231-82 ,eBioscience); TIGIT (Vstm3)-PE(Clone 4D4/ mTIGIT ,Cat#156103 ,BioLegend); CD160-PE(Clone 7H1 ,Cat#143003 ,BioLegend); NK1.1-PE/Cy5(Clone S17016D ,Cat#156523 ,BioLegend); PD-1-PE/Dazzle 594(Clone 29F.1A12 ,Cat#135227 ,BioLegend); CD69-PE/Dazzle 594(Clone H1.2F3 ,Cat#104535 ,BioLegend); CD107a (LAMP-1)-PE/Dazzle 594(Clone 1D4B ,Cat#121623 ,BioLegend); Ly-6C-PerCP/Cy5.5(Clone HK1.4 ,Cat#128011 ,BioLegend); CD62L-PerCP/Cy5.5(Clone MEL-14 ,Cat#104431 ,BioLegend); TIM3-PE/Cy7(Clone RMT3-23 ,Cat#119715 ,BioLegend); CD8a (clone D4W2Z, CST); GFP (clone D5.1, CST); PD-1 (EPR20665, Abcam); CK19 (EP1580Y.Abcam)
Validation	All antibodies used in this study were commercial antibodies and had been validated by the manufacturing companies. We provide the catalog number and supplier for each antibody.

Eukaryotic cell lines

Policy information about cell lines and Sex and Gender in Research

Cell line source(s)	HEK293T cell line was used for retrovirus production. Cell line were obtained from ATCC.
Authentication	The cell lines used in this study have been authenticated by STR profiling.
Mycoplasma contamination	Cell lines tested negative for mycoplasma.
Commonly misidentified lines (See ICLAC register)	No commonly misidentified cell lines were used in this study.

Animals and other research organisms

Policy information about studies involving animals; ARRIVE guidelines recommended for reporting animal research, and Sex and Gender in Research

Laboratory animals	IL-2Rα-/- (B6.129S4-Il2ratm1Dw/J), p40-/- (B6.129S1-Il12btm1Jm/J), CD4-/- (B6.129S2-Cd4tm1Mak/J), CD8a-/- (B6.129S2-Cd8atm1Mak/J), CXCR6GFP (B6.129P2-Cxcr6tm1Litt/J) and CD45.1 (B6.SJL-Ptprca Pepcb/BoyJ), PD-1-/- (B6.Cg-Pdcd1tm1.1Shr/J) on a C57BL/6J background were initially obtained from The Jackson Laboratory (Bar Harbor, ME, USA). The mice studied herein were maintained in individually ventilated cages under specific pathogen-free conditions in Laboratory Animal Center, South China University of Technology. These mice were maintained in 12h light/ 12h dark cycle, and the housing temperature and humidity were maintained 24°C and 50%.
Wild animals	no wild animals were used in the study
Reporting on sex	Both sexs of mice at 8-12 weeks of age.were used in the study.

Field-collected samples	no field collected samples were used in the study.
Ethics oversight	Animal experiments conformed to the guidelines outlined in the Guide for the Care and Use of Laboratory Animals, South China University of Technology.

Note that full information on the approval of the study protocol must also be provided in the manuscript.

Flow Cytometry

Plots

Confirm that:

☒ The axis labels state the marker and fluorochrome used (e.g. CD4-FITC).

☒ The axis scales are clearly visible. Include numbers along axes only for bottom left plot of group (a 'group' is an analysis of identical markers).

☒ All plots are contour plots with outliers or pseudocolor plots.

☒ A numerical value for number of cells or percentage (with statistics) is provided.

Methodology

Sample preparation	Liver was first homogenized with phosphate-buffered saline (PBS) containing 0.2% bovine serum albumin (BSA), passed through a steel mesh, and resuspended. Mononuclear cells (MNCs) from suspended liver cells were isolated by centrifugation with 40% Percoll (GE Healthcare, Little Chalfont, United Kingdom). Spleen was disrupted between two glass slides, suspended in PBS/0.2% BSA, and passed through a 70- micron nylon mesh. Red blood cells were depleted using RBC lysis buffer (Beyotime, China), and cells were suspended in PBs and counted on a hemocytometer in the presence of trypan blue.
Instrument	BD FACSAria II, BD LSRFortessa
Software	FACS data was anaylzed using FlowJO software v10.
Cell population abundance	FACS was performed by BD FACSAria II. The post-sorted population purity was tested by flow cytometry and over 95% purity. MACS was performed followed commercial kit's protocol. The post-sorted population purity was tested by flow cytometry and over 90% purity.
Gating strategy	Based on the pattern of FSC-A/SSC-A, living cells was gated with exclusion of dead cells and debris. Single cells were gated by FSC-A/FSC-H . Positive boundaries were etablished by using appropriate controls. The gating strategies show in the supplyment figures.

☒ Tick this box to confirm that a figure exemplifying the gating strategy is provided in the Supplementary Information.
